# First Draft Genome Assembly of Redlip Mullet (*Liza haematocheila*) From Family Mugilidae

**DOI:** 10.3389/fgene.2019.01246

**Published:** 2019-12-03

**Authors:** Dileepa S. Liyanage, Minyoung Oh, Welivitiye K.M. Omeka, Qiang Wan, Chang Nam Jin, Ga-Hee Shin, Byeong-Chul Kang, Bo-Hye Nam, Jehee Lee

**Affiliations:** ^1^Department of Marine Life Sciences and Fish Vaccine Research Center, Jeju National University, Jeju-si, South Korea; ^2^Research and Development Center, Insilicogen Inc., Yongin-Si, South Korea; ^3^Biotechnology Research Division, National Institute of Fisheries Science, Busan, South Korea; ^4^Marine Science Institute, Jeju National University, Jeju-si, South Korea

**Keywords:** draft genome, hybrid assembly, *Liza haematocheila*, ortholog and phylogenetic analysis, repeat analysis

## Introduction

**Figure 1:**
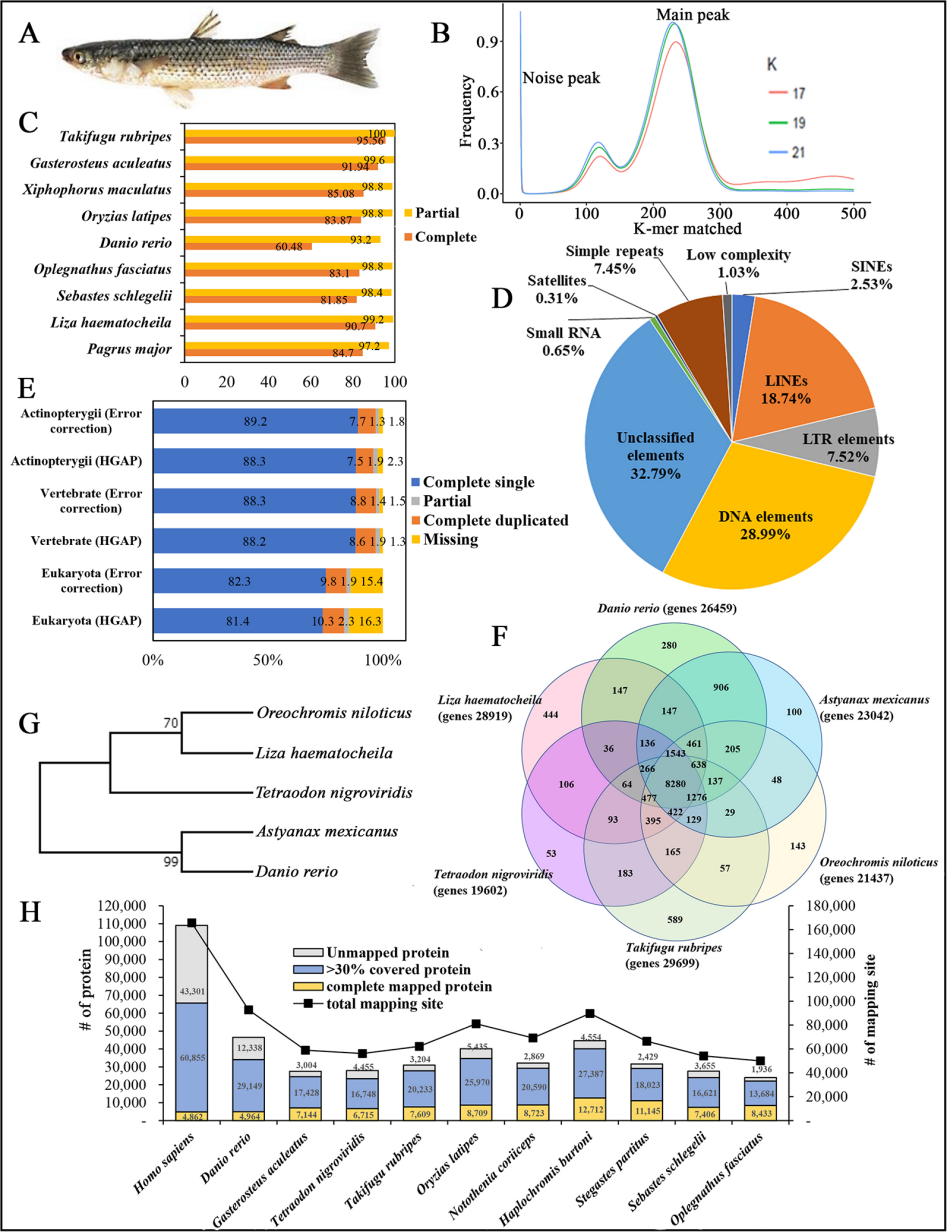
**(A)** Photograph of redlip mullet, *Liza haematocheila*. **(B)** Estimation of *L. haematocheila* genome size based on 17-, 19-, and 21-mer count analysis. **(C)** Genome assembly quality assessment from Core Eukaryotic Genes Mapping Approach (CEGMA). **(D)** Repeat elements of the hybrid draft genome. **(E)** Benchmarks of the Benchmarking Universal Single Copy Orthologs (BUSCO). **(F)** Venn diagram of orthologous gene clusters between six fish lineages. **(G)** Phylogenetic tree developed by maximum likelihoood method. **(H)** Mapped proteins and mapping sites of annotated genes from *Liza haematocheila* to other species.

Recent advances in next-generation sequencing (NGS) technologies have created opportunities to understand the genomic structure of higher-level organisms, and it has revolutionized the biological sciences and influenced aquaculture research. Discovery of marine organisms has progressed considerably due to NGS technologies, delivering a massive amount of genetic information quickly and affordably. Assembly of sequenced DNA reads to correct reference genomes is an essential task in genomic studies, for which diverse range of tools and algorithms have been developed. However, most of the genomes sequenced by NGS and other technologies are incomplete due to their large data size, time, and technical difficulties. High-throughput Illumina technology is exceptionally accurate, and thus, it is popular in the sequencing industry (Mavromatis et al., 2012). Also, single molecule real-time (SMRT) sequencing offered by Pacific Biosciences of California, Inc. (PacBio, USA) provides a longer average read length and high consensus accuracy (Yue et al., 2017); read length was nearly 27 kb under the PacBio Sequel System (Brown et al., 2014). Here, we present the draft genome of *Liza haematocheila* (*L. haematocheila*) (**Figure 1A**), which was sequenced using the current high-throughput sequencing platforms, PacBio and Illumina paired-end technologies, as a hybrid strategy.

Mullets belong to Family Mugilidae (Fish base ID: 359) and are globally distributed in tropical and temperate seas. They mainly inhabit in coastal and brackish waters, but some species, such as *Liza* abu, are found only in freshwater (Nelson et al., 2016). According to the literature, 30 genera and 78 mullet species have been identified to date, but the scientific nomenclature is a bit complex due to various synonyms (www.fishbase.de). Redlip mullet is identified by various synonyms, but the currently accepted scientific name is *L. haematocheila*. They are reported from Vietnam, Japan, and Korea, and have recently been introduced into the Aegean Sea, Black Sea, and the Mediterranean region (Streftaris and Zenetos, 2006; Hatooka et al., 2013; Papaconstantinou, 2014). Redlip mullet is an euryhaline species that migrates to the sea for breeding (Maurice Kottelat, 2008). They are a valuable fish species in Korea; they are cultivated along the west coast and account for 8% of the total fish supply in Korea (Han et al., 2015). Further, they have been used as a commercial food species for decades because of their wide salinity tolerance, adaptability to temperature fluctuations, high growth rate, high reproductive rate, and favorable taste (Meng et al., 2007). Due to their commercial importance, studies have investigated genetic variations of *L. haematocheila* based on various contexts, but a genome-wide study has not been conducted yet. Therefore, identification of genomic sequences, annotation of the genome, and a comprehensive genetic study is essential to identify and distinguish their genetic variation compared to other fish species; and it might be helpful for the further improvement of redlip mullet cultivation and breeding applications.

### Value of Data

– The genomic sequence data can be used for gene expression studies and future genetic breeding applications.
– The genomic sequence data were *de novo* assembled and annotated to create *Liza haematocheila* protein sequences.
– The draft genome of the *Liza haematocheila* was introduced for the first time and provides expansion in gene families of teleost.
– The developed draft genome can be used as a genomic reference for the discovery of genetic features, the advancement of marine science, and genome mining applications.

## Materials and Methods

### Sample Collection and DNA Extraction

Redlip mullet with an average weight and length of 100 g and 24 cm, respectively (NCBI taxonomy ID: 370040, FishBase ID: 13000), were obtained from the Sangdeok fishery in Hadong, South Korea and they were originated from south sea area (Sampling date, November 3, 2016). In order to determine the health conditions of the mullets, followed the guidelines for health and welfare monitoring of fish in the experiments (Johansen Needham and Colquhoun, 2006). High-quality genomic DNA (gDNA) was extracted from blood of a mullet using Qiagen MagAttract HMW DNA kit (Qiagen, Germany). For DNA quantification and purity, we used the Thermo Scientific™ Multiskan™ GO Microplate Spectrophotometer (Thermo Fisher Scientific, Inc., Waltham, MA, USA); and gDNA samples were sent to Insilicogen, Inc. (Yongin, South Korea) for NGS.

### Library Construction and DNA Sequence Data Generation

Before the library preparation, 0.6% TAE agarose gel was run to evaluate the DNA quality. The DNA sequencing was carried out with two libraries, with short and long reads. Qubit™ Fluorometric quantitation was performed to analyze the library concentration.

Longer read libraries (LRS, > 20 kb) were constructed for PacBio SMRT sequencing. A large amount of higher quality DNA (20 µg) was used for the LRS preparation, per the manufacturer protocols (PacBio, Menlo Park, CA, USA). Initially, gDNA was fragmented by Covaris g-TUBE™ (Covaris, Inc., USA) and size selection was made by more stringent AMPure^®^ PB product (Beckman Coulter Inc. USA). LRS templates were prepared with SMRTbell^®^ Express Template Prep kit (PacBio, Menlo Park, CA, USA). Then, ExoVII pre-treatment of DNA was carried out to remove the single-stranded ends from the sheared gDNA, following the repair DNA damages and end repair. Blunt hairpin adapters were ligated to the end-repaired DNA, and ExoIII/VII digestion was carried out to remove the failed ligation products. Adapter-ligated SMRTbell templates were purified using AMPure^®^ PB product. Prepared libraries were quality checked using a 2100 Bioanalyzer (Agilent Technologies, Inc., Santa Clara, CA, USA) and Qubit™ Fluorometric quantitation (Thermo Fisher Scientific, Inc., Waltham, MA, USA). Finally, sequencing primers were annealed to the quality-checked templates and sequenced on a PacBio Sequel System using one SMRT cell.

In addition to PacBio reads, we employed Illumina paired-end sequencing by synthesis (SBS) technology to sequence the redlip mullet genome using the whole genome shotgun (WGS) approach. 2 µg of mullet gDNA were fragmented, and size selected, using the same protocol described above. Paired-end short read sequencing (PE-SRS) libraries (Illumina HiSeq 2500 System, 2 × 250 bp) were prepared with Illumina HiSeq Rapid v2 SBS Kits with an average insert size of 550 bp. PE-SRS libraries were sequenced on an Illumina HiSeq 2500 System under the rapid run mode, per standard Illumina protocols (Illumina, Inc., San Diego, CA, USA).

### Sequence Data Processing and Genome Assembly

Draft genome sequence data, generated using an Illumina 550 bp PE-SRS library, generated 788,518,494 total raw reads; 723,780,592 total high-quality reads (Phred quality score > Q20 and >100 × coverage); and 162,861,516,777 total sequence data, after cleaning and removing contaminations. After generating the paired-end reads, we performed data filtering and adapter trimming, to remove PCR duplications and adapter contamination, using Trimmomatic 0.38 (http://www.usadellab.org/cms/index.php?page=trimmomatic) (Bolger et al., 2014), with parameters ILLUMINACLIP: TruSeq3-PE-2.fa:2:30:10 LEADING:3 TRAILING:3 SLIDINGWINDOW:4:20 MINLEN:30. Contaminates (bacterial draft sequences, bacterial sequences, and virus sequences) were removed with contam DB ocean meta (NCBI WGS project: AACY02) (Venter et al., 2004) (**Table 1A**).

**Table 1:** Summary statistics of *Liza haematocheila* draft genome.

(A) Sequencing reads	Illumina HiSeq PE	PacBio Long read
Raw data (coverage)	197.9 Gb (∼217X)	71.1 Gb (∼94X)
Pre-processed data (%)	162.86 Gb (82.2)	70.9 Gb (99.6)
**(B) Assembly data**		
No. contigs	1,453	
Residues	747,342,578 bp	
Avg. length	514,344 bp	
Min. length	151 bp	
Max. length	20,110,137 bp	
N50	3,973,280 bp	
N (%)	0.0	
GC (%)	42.42	
**(C) Genetic elements**		
# of tRNA	2,457	
# of rRNA	903	
# of genes	28,919 (6.79 exons/gene)	
Avg. gene length	7,719 bp	
Avg. exon length	170 bp	
Repeat elements	29.27%	
Genome coverage (gene region)	29.87%	
**(D) Annotation**		
Blast hits (%)	21,796 (75.37)	
No hits (%)	7,123 (24.63)	
GO annotated (%)	20,310 (70.23)	
**(E) Top 10 GOs**		
**Biological process**	**Cellular component**	**Molecular function**
Biological process (1,726)	Membrane (6,571)	Molecular function (1,979)
Transport (1,406)	Integral component of membrane (6,010)	Metal ion binding (1,964)
Regulation of transcription, DNA-templated (1,373)	Nucleus (3,012)	ATP binding (1,625)
Signal transduction (1,224)	Cytoplasm (2,545)	Transferase activity (1,611)
Transcription, DNA-templated (830)	Cellular component (2,025)	Nucleotide binding (1,407)
Phosphorylation (753)	Plasma membrane (1,430)	DNA binding (1,398)
Protein phosphorylation (737)	Integral component of plasma membrane (835)	Zinc ion binding (1,162)
Ion transport (701)	Extracellular region (698)	Hydrolase activity (1,123)
G-protein coupled receptor signaling pathway (660)	Intracellular (495)	Nucleic acid binding (1070)
Multicellular organismal development (643)	Mitochondrion (470)	Calcium ion binding (841)

After sequencing on PacBio, analysis of raw reads, which contained the barcode and adapter duplicates, was performed on the SMRT Link Data Management portal (v 5.1), using subread filtering protocol. The PacBio library on one SMRT cell produced 6,856,161 polymerase reads and 8,082,917 subreads, with 71,185,126,879 and 70,938,377,243 bases, respectively, with a 17.7 kb polymerase read N50 and 13.8 kb subread N50 (**Table 1A**).

K-mer analysis for the redlip mullet genome was conducted with Jellyfish 2.2.3 software (http://www.cbcb.umd.edu/software/jellyfish/). The frequencies of different k-mers (17-, 19-, and 21-mers) and their sequence depths were plotted as k-mer frequency distributions. Main peak in the K-mer frequency distribution graph gives matched k-mer value for genome size estimation. Noise peaks were formed by very low coverage error k-mers (noise) from sequencing errors. The highest peak was observed at estimated depths of 233, 231, and 228 for the 17-, 19-, and 21-mers, respectively (**Figure 1B**). Repeated sequences in the genome affected the shape of the k-mer distribution curve, depending on the genome length and copy number. Therefore, repeated sequences in the genome produced higher abundances of associated k-mers. The peak distributions indicated that the genome is slightly repetitive, heterozygous, and diploid. The genome size of *L. haematocheila* was estimated by dividing the number of k-mers from the volume of the peak in the k-mer frequency distribution. According to the k-mer size estimations, similar genome lengths (698–714 Mb) were observed, and the estimated genome size of *L. haematocheila* was 700 Mb. We generated more than 30× coverage of the redlip mullet genome using one SMRT cell.

PacBio SMRT sequence reads were assembled with Hierarchical Genome Assembly Process (HGAP). Briefly, low-quality reads were filtered from the raw reads. Then, filtered subreads, shorter than 11 kb, were identified as short reads and others separated as long reads. Short reads were preassembled by aligning them to longer seeding sequences, using Basic Local Alignment with Successive Refinement (BLASR) (https://github.com/PacificBiosciences/blasr). Preassembled reads were aligned to the obtain longer reads with a PBcR assembly program (Celera Assembler) (http://wgs-assembler.sourceforge.net/wiki/index.php?title=Main_Page) (Berlin et al., 2015). Assembly polishing was conducted with Quiver (https://github.com/PacificBiosciences/GenomicConsensus) to exclude the insertion-deletion events and single nucleotide polymorphisms in the draft assembly (Chin et al., 2013). Recently, different genome techniques were merged to develop *de novo* assembly with high coverage. Therefore, Illumina paired-end reads were used to correct the errors in the SMRT reads (Koren et al., 2012; Hackl et al., 2014; Salmela and Rivals, 2014).

The HiSeq paired-end reads were mapped to the draft genome assembly generated from the PacBio reads. The error corrected PacBio + HiSeq draft assembly contained total 1453 contigs with ∼747 Mbp and contig N50 length was ∼3.9 Mb (**Table 1B**). As redlip mullet has an uncurated draft genome, we relied on the assembly statistics of other fish genomes as follows: The *de novo* assembly for *Salmo salar* produced 2,966 Mbp, 57 kb contig N50, and 368,060 contigs with HiSeq, PacBio, Sanger, and GAIIx technologies sequencing methods (https://www.ncbi.nlm.nih.gov/assembly/GCF_000233375.1). *Oncorhynchus mykiss* produced 2,178 Mbp, 13 kb contig N50, and 559,855 contigs with Illumina HiSeq sequencing data (https://www.ncbi.nlm.nih.gov/assembly/GCF_002163495.1). Paralichthys olivaceus produced 545 Mbp, 5 kb contig N50, and 38,614 contigs with Illumina sequencing data (https://www.ncbi.nlm.nih.gov/assembly/GCA_001904815.2). *Danio rerio* produced 1,373 Mbp, 1.4 Mb contig N50, and 19,725 contigs with multiple sequencing methods (https://www.ncbi.nlm.nih.gov/assembly/GCF_000002035.6). Therefore, the hybrid assembly approach used in this study was considered effective in reducing the number of contigs and increasing the contig N50 length of the heterozygous diploid genome, by combining Illumina HiSeq short-read and PacBio long-read sequences.

The quality and completeness of both assemblies (HGAP and error corrected using short reads) were assessed using Core Eukaryotic Genes Mapping Approach (CEGMA) v. 2.5 (http://korflab.ucdavis.edu/datasets/cegma/) (Parra et al., 2007) with EuKaryotic Orthologous Groups (KOG) datasets. CEGMA revealed 248 core genes; 90.7% complete and 99.2% partial core eukaryotic genes were mapped to our assembly (**Figure 1C**). Further, Benchmarking Universal Single Copy Orthologs (BUSCO) v. 2.0 notation scores analysis (http://busco.ezlab.org/) (Waterhouse et al., 2018) was used assess the quality of the assembled sequence and the predicted protein sequences of the redlip mullet draft genome, compared to reference genome datasets of Eukaryote, Vertebrate, and Actinopterygii. According to the BUSCO analysis, the redlip mullet draft genome comprised 82.3%, 88.3%, and 89.2% similarity with the Eukaryote, Vertebrate, and Actinopterygii datasets, respectively for complete single sequences with error correction (**Figure 1E**).

### Repeat Analysis, Gene Prediction, and Gene Ontology (GO) Annotation

Genomic repeat elements were identified by RepeatMasker (v4.0.5) (http://www.repeatmasker.org/) using RMBlastn v2.2.27 and Repbase v20.08 database (https://www.girinst.org/repbase/). 1,453 sequences with 747,342,578 bp were submitted to RepeatMasker and among them, 218,396,110 bases were masked with 29.22% of the *L. haematocheila* genome which observed as repeats (**Figure 1D**). The GC content of the redlip mullet draft genome was 42.43%. Short interspersed nuclear elements (SINEs) accounted for 0.74% of the assembled draft sequence, and long interspersed nuclear elements (LINEs) accounted for 5.48%. Regarding non-coding DNA sequences of SINE elements, Alu and mammalian-wide interspersed repeats comprised 0.002% and 0.08% of the genome sequences, respectively. Non-long terminal repeat (LTR) retrotransposable elements in LINEs identified as LINE1, LINE2, and L3-Chicken repeat 1 accounted for 0.62%, 1.77%, and 0.01% of the sequence, respectively. Retrotransposable elements in LTRs accounted for 2.20%, where endogenous retrovirus classes I and II were observed as the dominant types in the draft genome sequence. DNA transposable elements, like hAT-Charlie and TcMar-Trigger, occupied a substantial percentage (8.48%) of the genome. Simple repeat sequences (microsatellites, simple sequence repeats, or short tandem repeats) accounted for 2.18%, and satellite sequences comprised 0.09%.

Prediction of protein-coding genes was carried out with the integration of three different prediction strategies: mRNA, protein, and *ab initio*. For transcript-based prediction (mRNA model), Isoform- and RNA-sequencing data from mullet tissues were mapped using TopHat v2.1.1 (http://ccb.jhu.edu/software/tophat/index.shtml) (Trapnell et al., 2012). Gene and transcript expression was analyzed using Cufflinks v2.2.1 (http://cole-trapnell-lab.github.io/cufflinks/cuffmerge/) (Trapnell et al., 2012). For the protein-based homology analysis, 11 diverse organisms (*Homo sapiens, Denio*. rerio, *Gasterosteus aculeatus*, *Tetraodon* nigroviridis, *Takifugu* rubripes, *Oryzias* latipes, *Notothenia* coriiceps, *Haplochromis* burtoni, *Stegastes* partitus, *Sebastes* schlegelii, and *Oplegnathus* fasciatus) were aligned by NCBI tblastn (e-value 10,000; matrix BLOSUM62; word_size 3). To predict the genes in the draft genome sequence, three different strategies were used. Initially, *Ab initio* gene prediction was performed with the training data set using the AUGUSTUS gene prediction program (http://bioinf.uni-greifswald.de/augustus/). Also, mRNA hidden Markov model was applied to predict the splice donor, splice acceptor, coding exon state, intron state, and initiation termination codons (Stanke et al., 2006). Further, GeneID gene prediction software (http://genome.crg.es/software/geneid/) was used to predict the splice sites, start and stop codons, and exons using the parameter file for *Tetraodon* nigroviridis (Parra et al., 2000) on repeat masked assembly. *St*. partitus and *Op*. fasciatus showed the highest mapped rates of 61.83% and 61.62% of proteins to *L. haematocheila*, respectively. The highest unmapped rate of proteins was observed in *H. sapiens* (**Figure 1H**). Genetic elements were observed as 2,459 tRNAs, 903 rRNAs, and 28,919 total annotated genes with 6.79 exons per gene. Additionally, an average gene length of 7,719 bp and an average exon length of 170 bp were observed in the annotated draft genome of redlip mullet. Genomic coverage of the gene regions was 29.87%, with 29.27% repeat elements (**Table 1C**).

Final consensus transcripts were obtained from the gene models subjected to gene ontology (GO) analysis using Blast2Go software (http://www.blast2go.com/b2ghome) (Gotz et al., 2008). Out of all annotated genes, there were 2,796 (70.23%) blast hits found using the National Center for Biotechnology Information (NCBI) non-redundant database (e-value 0.00001). GO categories of biological function, cellular component, and molecular function were annotated from identified genes (**Tables 1D, E**). One hundred sixteen of Kyoto Encyclopedia of Genes and Genomes (KEGG) pathways (https://www.genome.jp/kegg/pathway.html) were identified from the final consensus transcripts, and 61 enzymes were found in the KEGG metabolic pathway maps for the biosynthesis of antibiotics, 29 enzymes for purine metabolism and 19 enzymes for glycolysis/gluconeogenesis.

### Ortholog and Phylogenetic Analysis

Ortholog analysis was conducted using OrthoMCL v2.0.5 (http://www.orthomcl.org/cgi-bin/OrthoMclWeb.cgi) (Li et al., 2003). We used default parameters specified with OrthoMCL to run the six protein data sets from *L. haematocheila*, *D*. rerio, *Astyanax* mexicanus, *Oreochromis* niloticus, *Ta*. rubripes, and *Te*. nigroviridis. The species formed 21,430 clusters, 19,821 orthologous clusters (containing at least two species), and 5,197 single-copy gene clusters. 8,280 orthologous groups were identified as common in all the selected fish species, and 444 groups specific to *L. haematocheila* were not identified in the others (**Figure 1F**).

The phylogenetic tree was constructed to show the ortholog relationship, which was already simulated by ortholog analysis. Initially, one to one protein-coding orthologs were identified using OrthoMCL v2.0.5. They were aligned using the multiple alignment program for amino acid or nucleotide sequences—MAFFT v7.45 software (https://mafft.cbrc.jp/alignment/software/linux.html). Poorly aligned positions and divergent regions of DNA alignment were filtered out by using Gblocks v0.91 (http://molevol.cmima.csic.es/castresana/Gblocks.html). After merging the filtered alignments, maximum likelihood method was applied to draw the phylogenetic tree using RAxML v8.0.0 (https://cme.h-its.org/exelixis/web/software/raxml/index.html) (**Figure 1G**).

In summary, we report the first draft genome of *L. haematocheila*. We hope this study provides useful information for future studies. Since there are limited genomic sequence resources published about marine organisms, our study provides more insights into the teleost genome discoveries.

### Code Availability

In this study, we did not use any custom codes. However, according to software-specific recommendations, the default parameters were changed intentionally to obtain better results. Those parameters were provided in the text along with the relevant software or tool. We used Linux to execute the commands.

## Data Availability Statement

This Whole Genome Shotgun sequencing project has been deposited at DDBJ/ENA/GenBank under the accession SRSE00000000.1, BioProject ID PRJNA531337, BioSample SAMN11356700. The version described in this paper is version SRSE01000000 and data can be accessed at NCBI (https://www.ncbi.nlm.nih.gov/assembly/GCA_005024645.1).

## Ethics Statement

This study was carried out in accordance with the recommendations of ‘Animal Ethics Committee of Jeju National University’. The protocol was approved by the ‘Jeju National University’.

## Author Contributions

JL, MO, QW, CJ, and B-HN designed and conducted the study. G-HS and B-CK performed the sequencing and genome assembly. G-HS, B-CK, and DL performed data analysis. DL, WO, and JL wrote the manuscript.

## Funding

This research was supported by a grant from the Marine Biotechnology Program (20180430, Genome Analysis of Marine and Fisheries Organisms and Development of Functional Application), funded by the Ministry of Oceans and Fisheries, Korea.

## Conflict of Interest

Authors G-HS and BC-K were employed by company Insilicogen Inc., Korea.

The remaining authors declare that the research was conducted in the absence of any commercial or financial relationships that could be construed as a potential conflict of interest.
